# Incidence of HIV Infection among HIV-Exposed Iinfants at Gondar University Hospital from 2019-2021: A Prospective Cohort Study

**DOI:** 10.4314/ejhs.v33i2.5

**Published:** 2023-03

**Authors:** Mehretie Kokeb

**Affiliations:** 1 Department of Pediatrics and child Health, University of Gondar, Ethiopia

**Keywords:** HIV-exposed, Infants, Incidence, HIV Infection, Gondar

## Abstract

**Background:**

Mother-to-child transmission (MTCT) of human immunodeficiency virus (HIV) is decreasing worldwide; however, achieving the MTCT elimination target of 2% by 2020 and 0% by 2030 is challenging in resource-limited countries. Preventing mother-to-child transmission (PMTCT) is a key strategy in eliminating new pediatric human immunodeficiency virus (HIV) infection Strengthening PMTCT program is one of the key mechanisms for the elimination of Pediatric HIV infection and improving maternal and newborn survival. Assessing the incidence of HIV infection among HIV exposed infants is critical to devise an important preventive strategy which was the main objective of this study.

**Methods:**

A prospective Cohort study was conducted at Gondar University Comprehensive Specialized Hospital, PMTCT clinic to assess the incidence of HIV infection among HIV exposed infants from 2019-2021.

**Results:**

The overall incidence of HIV infection among HIV exposed infants was 3.6%. HIV infection rate was significantly increased among HIV exposed infants coming out of Gondar, Infants with developmental failure and Infants with Unknown fathers' HIV status compared to their counterpart.

**Conclusions:**

The incidence of HIV infection among 307 HIV-exposed infants was 3.6% which is higher than the expected standard. The lost to follow up rate was also significant (9.4%). These finding showed that strengthening the PMCT service is mandatory.

## Introduction

Mother to Child Transmission of HIV (MTCT) is the transmission of HIV from an HIV-positive mother to her child during pregnancy, delivery or through breastfeeding contributing for more than 90% of HIV infections in infants. Even if PMTCT has been implemented worldwide, HIV-infection rate among HIV-exposed infants is still unacceptably high especially in developing countries (1).

Ethiopian federal ministry of health (FMoH) implemented the PMTCT program in 2011 to provide effective interventions on MTCT of HIV during pregnancy, labor and delivery, and the breastfeeding period by providing ARV drugs for the mother and the baby. In addition, an option B+ approach which is immediate initiation of lifelong ART for all pregnant or breast-feeding mother regardless of clinical stage and CD4 count had been implemented in 2013. Similar programs were also launched in many African countries with different reports of HIV infection rate among HIV exposed infants (2,3).

A report from Nigeria revealed an incidence of HIV infection among HIV exposed infants to be 2.8% which is nearer to the WHO indicator of below 2% and this study reported the Lost to Follow Up(LTFU) rate of 7.3% (LTFU) and rural maternal residence, lack of maternal ART/ARV prophylaxis, mixed infant feeding and infant birth weight less than 2.5 kg were found to have correlation with risk of HIV-infection in HIV exposed infants (4). A study done in 5 Amhara regional state, Ethiopia, referral Hospitals to assess attrition rate (Death, Lost to follow up) of HIV exposed infants reported that the cumulative incidence of LTFU was 19 (8.8%), (95% CI, 5.4%-12.4%) among 217 exposed infants enrolled to the PMTCT department between January 2014 and 2017 and the cumulative incidence of mortality in the region was zero (5). A systematic review and meta-analysis done in low- and middle-income countries to evaluate the attrition of HIV-exposed infants from early infant diagnosis services showed that Overall attrition (LTFU + death) from EID services was 25% (95% CI: 20% to 29%) by two months of age and increased to 39% (95% CI: 27% to 50%) by 18 months of age. LTFU accounted for the preponderance of overall attrition, with 17% (95% CI: 13% to 21%) of HIV exposed infants LTFU by two months of age and 26% (95% CI: 21% to 32%) LTFU by18 months of age. Death accounted for a smaller proportion of overall attrition, with prevalence ranging from 3% (95% CI: 2% to 3%) by two months of age to 6% (95% CI: 5% to 7%) by 18 months of age (6).

Another study done in Dessie, Ethiopia, reported HIV-infection rate 3.8% of among HIV exposed infants and absence of maternal antenatal care visit (AOR = 4.6, 95% CI: 1.17–17.99), home delivery (AOR = 4.2, 95% CI:1.04 16.76), absence of antiretroviral intervention to the mother (AOR= 5.7, 95% CI:1.10–29.36), and failure to initiate nevirapine prophylaxis for the infant (AOR = 5.3, 95% CI: 1.11 -25.44) were found to be significant factors.

Gondar University Comprehensive Specialized Hospital is the only referral hospital in the area where HIV-exposed infants have being getting care for more than 15years but there are scarcity of evidences about the incidence of HIV infection among HIV-Exposed infants and the already documented facts were done retrospectively and dated more than 7years which was the main rationale of this study which is a prospective follow up study done over 3years period.

The main objective of this Prospective Cohort study was Study to assess the Incidence of HIV Infection among HIV-Exposed infants at Gondar University Comprehensive Specialized Hospital PMTCT clinic from 2019–2021, Northwest Ethiopia:

## Methods and Materials

**Study design and period**: A prospective cohort study was conducted to assess the incidence of HIV infection among HIV-exposed infants at Gondar University Comprehensive Specialized Hospital PMTCT clinic from 1^st^ January, 2019–31^th^ January, 2021.

**Study area:** This prospective Cohort study was conducted at Gondar University Comprehensive Specialized Hospital (GUCSH), PMTCT clinic. The Hospital is Located in Gondar town 741km Northwest of the capital city Addis Ababa. GUCSH is one of the oldest Hospitals in Ethiopia and has been giving HIV/AIDS care since 2005. Annually; there are nearly 600 HIV infected children below 15years of age and 150 HIV-Exposed infants having follow-up at GUCSH.

**Study population**: The study population of our study was all HIV-exposed infants enrolled at GUCSH PMTCT clinic from 1^st^ January, 2019–31^th^ January, 2021.

**Sample size determination**: The samples size, 322, was calculated using a single population proportion formula. The following assumptions were considered in calculating the sample size: CI = cumulative incidence of HIV infection among HIV-exposed infants done in Southwest Ethiopia was 17%, [Za/2 = Z score of 95% CI, d =margin of error (4%precision), and non-response rate of 10% (20).

**Inclusion and exclusion criteria**: All HIV-Exposed infants age below 18months who had follow up during the study period were included with no exclusion.

**Operational definitions**:

**HIV-Exposed infant:** A child aged below 18 months and born to HIV positive mother OR HIV antibody test positive

**Lost to Follow-up:** Those who didn't come for subsequent 2 follow up schedules and failed to be traced

**End of follow-up**: Those lost or died before knowing HIV infection status

**Discharged:** Those sent home after 18months with confirmation of negative HIV test

**Data collection procedure**: Data was collected by Nurses and Residents working in the PMTCT clinic by using a structured data abstraction checklist from HIV-Exposed infants till the end up of their follow up. An infant identified as being HIV-exposed from different sites (delivery ward, Pediatric OPD or other health institutions) and referred to PMTCT clinic of GUSCH were enrolled and every infant was followed until HIV status is confirmed as positive or negative. Those identified as HIV infected were linked to ART clinic immediately and others were followed till completing their 18months of follow-up.

Data were collected directly from the caretakers and whenever needed medical records were revised. A total of 322 HIV-exposed infants were identified during the study period but 15 were not included in the analysis because caregivers declined their consent. The follow up was done from 1^st^ January 2019 to 30^th^ June 2022. The enrollment was continued till 31^st^ January 2021 and the last follow up was done on 30^th^ June 2022 making exactly 18months from the last date of enrollment. All variables including socio-demographic, parental conditions, clinical and laboratory parameters were completed when the infant completes the follow up. Data was checked for inconsistency, accuracy, clarity and completeness and corrections were given on each case.

**Data processing and analysis**: Data were cleaned, coded and entered in to Epi Data Version 3.1 after correct entry was checked then data were exported to SPSS version-26 software for analysis. Further data accuracy and missing values were checked on SPSS. Both descriptive and analytical statistical procedures were utilized. Descriptive statistics like percentage, mean, median and standard deviations were used for the presentation of characteristics of HIV-Exposed infants. Tables and figures were used for data presentation. To identify the factors associated with the incidence of HIV infection among HIV-Exposed infants, bivariate and multi-variate logistic regression analysis techniques were applied. The results of the regression analysis were reported as OR with their 95% confidence interval and p-value. For the Bivariate regression, variables with p value<0.2 were entered in to multivariate logistic regression and p-value <0.05 was considered statistically significant.

**Ethical considerations**: Ethical clearance was obtained from Institutional Review Board of University of Gondar. Informed consent was given for every infant's caretaker and all the necessary explanations about the purpose of the study and its procedure was explained to them with the assurance of confidentiality of the information collected and informing them about their right to withdraw at any time during the follow up period from the study. Privacy of the patients was maintained by not including names, using codes and questionnaires were kept locked.

## Results

In this study, a total of 307 HEIs were included (47.6%) were females and most of them were from Gondar town (75.9%). The majority (76.2%) of HIV-exposed infants was delivered at health institution and the remaining 23.8% of them were home deliveries. The care takers were mother, father and relative in 98.7%, 1% and 0.3%, respectively. Four (1.3%) mothers were reported to be died and 13(4.2%) fathers were died (Table 1).

**Table 1:** Socio-demographic characteristics of HIV-exposed infants

Characteristics	Frequency (%)
**Sex**	
Female	146 (47.6)
Male	161 (52.4)
**Address**	
Gondar	233 (75.9)
Out of Gondar	74 (24.1)
**Place of Delivery**	
Health Institution	234(76.2)
Home	73 (23.8)
**Care Taker**	
Mother	303(98.7%)
Father	3(1)
Relative	1(0.3)
**Mother**	
Alive	303(98.7)
Died	4(1.3)
**Father**	
Alive	294(95.8)
Died	13(4.2)

**Clinical and laboratory profiles of parents**: Out of 307 mothers, 219(71.3%) were started on ART before pregnancy and the remaining 88(28.7%) were not on any form of HIV treatment before pregnancy but during the PMTCT follow up 77(87.5%) of them were started on ART. Maternal CD4 during pregnancy were reported as above 350 in 250(81.4%), below 350 in 49(16%) and Unknown in 8(2.6%) of mothers, respectively. Majority (47.6%) of fathers were not tested for HIV and among tested fathers, 44 (14.3%) were reported to be negative (Table 2).

**Table 2:** Clinical and laboratory findings of parents of HIV-exposed infants

Characteristics	Frequency (%)
**Mother Started on ART** **before pregnancy**	
Yes	219(71.3)
No	88(28.7)
**Mother started ART at PMTCT**	
Yes	77(87.5)
No	11(12.5)
**Maternal CD4 before Birth**	
≥350	250(81.4)
<350	49(16)
Unknown	8(2.6)
**Father's HIV status**	
Negative	44(14.3)
Positive	117(38.1)
Unknown	146(47.6)

**Clinical and laboratory Profiles of HIV-exposed infants**: Among 307 HIV-Exposed (HEI) infants, 234 (76.2%) were on infant prophylaxis but 73 (23.8%) were not on any form of prophylaxis for HIV. Feeding practice of HIV-exposed infants before 6months of age showed that 219(71.3%), 41(13.4%) and 47(15.3%) were on Exclusive feeding, Replacement feeding and mixed feeding, respectively. Clinical profiles of infants during follow up revealed that 85(27.7%) were having growth failure and 46 (15%) were found to have developmental failure. Laboratory findings reported that DNA PCR test was done in majority 298 (97.1%) of HIV-exposed infants with positive rate of 8(2.7%). Antibody test was done on a total of 251(81.8%) of infants at 18months of age with positive report of 3(1.2%) on whom DNA PCR was not done (Table 3).

**Table 3:** Clinical and laboratory findings of HIV-exposed infants

Characteristics	Frequency
**Infant's Prophylaxis**	
Yes	234(76.2)
No	73(23.8)
**Feeding (1^st^ 6months)**	
Exclusive Breast feeding	219(71.3)
Replacement Feeding	41(13.4)
Mixed Feeding	47(15.3)
**Growth Failure**	
Yes	85(27.7)
No	222(72.3)
**Developmental Failure**	
Yes	46(15)
No	261(85)
**DNA PCR test**	
Done	298(97.1)
Not done	9(2.9)
**DNA PCR test result**	
Negative	290(97.3)
Positive	8(2.7)
**Antibody test (at discharge)**	
Done	251(81.8)
Not Done	56(18.2)
**Antibody test result (at** **18months of age)**	
Negative	248(98.8)
Positive	3(1.2)

**Figure 1:**
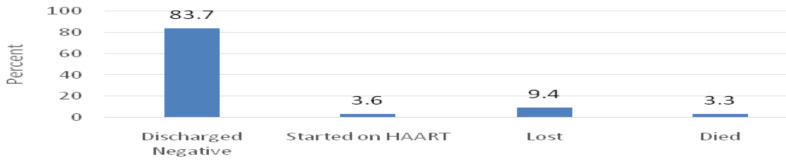
Final Outcome of HIV Exposed Infants.

**Final outcome**: At the end of the follow up of the 307 HIV-Expose infants: 257(83.7%) , 29(9.4%), 11 (3.6%) and 10 (3.3%) were discharged negative, lost to follow up, confirmed HIV inefected and died before knowing their HIV status, respevtively (Figure 1).

There were a total of 11 HIV-exposed infants who ended up with HIV infection during the 3years follow up period; 8were diagnosed by DNA PCR and only 3were confirmed by HIV antibody test. Six of them were having infant ARV prophylaxis and 4 mothers were not on ART before delivery. Majority (5/11) of HIV infections were recorded in 2019 and the remaining 6 HIV infections were reported in the years 2020 and 2021, 3cases in each year (Table 4).

**Table 4:** Overall characteristics of the 11 HIV infected children

Age (in months) at enrollment	Mother started ART (duration before delivery)	ARV prophylaxis given to the infant	HIV infection confirmed at the age of	Type of HIV test	Year
6months	No	No	9months	DNA PCR	2019
At birth	At time of delivery	Yes	8weeks	DNA PCR	2019
At birth	6months	Yes	8months	DNA PCR	2019
9months	5years	No	18months	Antibody	2019
At birth	2years	Yes	3months	DNA PCR	2019
11months	No	No	19months	Antibody	2020
At birth	4months	Yes	6months	DNA PCR	2020
At birth	8years	Yes	9weeks	DNA PCR	2020
6weeks	3years	No	10weeks	DNA PCR	2021
4months	1year	No	18months	Antibody	2021
At birth	At time of delivery	Yes	8weeks	DNA PCR	2021

**Predictors of MTCT**: To understand the factors predicting MTCT, possible maternal and infant predictors were first analyzed individually using a bivariate logistic regression; Address, Maternal PMTCT enrollment, Infant's developmental failure, Maternal CD4 before birth, place of delivery and Father's HIV status were found to have significant association with final HIV status of HIV-Exposed infants. All the variables that were significant at the 0.2 level in the bivariate analysis were included in the multivariate logistic regression model and stepwise method of model selection was used to identify a subset of these risk factors that were independently associated with MTCT. Adjusting for other factors, Address, Infant's developmental failure and Father's HIV status were independent predictors of HIV Infection among exposed infants. The overall incidence HIV infection among HIV exposed infants was 3.6%. HIV infection rate was significantly increased among HIV exposed infants coming out of Gondar (AOR=4.9, 95%CI: 1.765, 35.80), Infants with developmental failure (AOR=3.57, 95%CI: .032, 7.58) and Infants with Unknown fathers' HIV status (AOR=1.78, 95% CI: .028, 12.035) compared to their counterpart (Table 5).

**Table 5:** Associated factors with final HIV infection of HIV-exposed infants

*Variables*	*COR (95% CI)*	*P-Value*	*AOR (95% CI)*
***Address***	***2.11 (0.062 4.720)***	***.007***	***7.950 (1.765, 35.801)***
**Mother enrolled to PMTCT**	0.271 (0.080, 0.917)	.292	2.867 (0.404, 20.327)
***Developmental Failure***	***4.868 (1.338, 17.711)***	***.021***	***3.57 (0.032, 7. 58)***
**Maternal CD4 before birth**	1.561 (0.440, 8.461)	.301	0.271 (0.023, 3.221)
**Place of Delivery**	0.792 (0.202, 3.106)	.794	0.776 (0.116, 5.209)
**Father's HIV status**	**2.621 (0.773, 8.884)**	**.004**	**1.78 (0.028, 12.035)**

COR=Crude Odds Ratio AOR=Adjusted Odds Ratio

## Discussion

Our prospective cohort study was done to assess the incidence of HIV infection among 307 HIV exposed infants at the University of Gondar Hospital over 3years (2019–2021) period. To understand the predictors of MTCT; possible maternal and infant predictors were first analyzed individually using a bivariate logistic regression by which Address, Maternal PMTCT enrollment, Infant's developmental failure, Maternal CD4 before birth, place of delivery and father's HIV status were found to have significant association with final HIV status of HIV-Exposed infants. But during the multivariate logistic regression analysis only Address, Infant's developmental failure and father's HIV status were independent predictors of HIV Infection among exposed infants. This might be explained by the fact that mothers under proper care could have delivered at home and visited the hospital for the infants care and maternal CD4before birth is not significant during multivariate analysis which might be explained by the fact that other factors in the mother such us mode of delivery and viral load could have masked the effect of CD4 count on rate of HIV-infection among HIV-exposed infants.

The incidence of HIV infection among HIV exposed infants was found to be 3.6% (11/307). Our prospective cohort study also showed a LTFU rate of 9.4% (29/307) and a death rate of 3.3% (10/307) among HIV exposed infants. Using multiple logistic regression analysis; infants coming out of Gondar were 7.95 times at higher risk of HIV infection compared to those coming from Gondar (AOR=4.9, 95%CI: 1.765, 35.80). Infants with developmental failure were 3.6 times at risk of HIV infection in comparison with infants without developmental failure (AOR=3.57, 95%CI: .032, 7.58) and Infants with Unknown fathers' HIV status were 2 (AOR=1.78, 95% CI: .028, 12.035) times at more risk of HIV infection compared to infants with known fathers' HIV status. This finding is lower than a report from Vietnam which showed 8.9% (47/472) rate of MTCT and absence of maternal ART and ARV prophylaxis were significant contributors of HIV infection (9). Another study from Harare, Zimbabwe done in 2017–2018 reported 1.55% rate of MTCT and mortality rate of 0.88% which is lower than the report of our study (10). The higher rate of transmission in Vietnam study was explained due to the fact that most of the study participants were taken before the implementation of Option B+ and the significant lower rate of HIV infection rate in Zimbabwe was due to the fact that the research was done among pregnant mothers on follow up where all enrolled mothers were on PMTCT intervention which is in contrast with our study participants who were infants presenting to PMTCT clinic in the 1st 18months of age so that infants presenting late were not having any prophylaxis which in turn increases rate of HIV infection.

Similar reports from Ethiopia done from 2011–2015 showed an average HIV-infection rate of 5–8% among HIV exposed infants which is higher than our report of 3.6% and absence of maternal ART, absence of Infant ARV prophylaxis and home delivery were major contributors (11–15). This is explained by the timing of researches and differences of the setups where our study was done in a tertiary Hospital whereas others were done in primary Hospitals. A systematic review done in Ethiopia from 2010–2017 by Kassa G. revealed that there was an average MTCT rate of 10% ranging from 4,16% to 15.7% and similar to the other reports; absence of maternal ART, home delivery and absence of infant ARV prophylaxis were associated factors. This difference was explained by the difference in setups and time of study which is also having similar explanation for the differences with our study (16). Another systematic review from different regions of Ethiopia reported a MTCT rate of 4.4% and rural residency, home delivery and absence of ART to mothers were significant independent factors (17) and similar systematic review done from 2005 to 2015 reported postnatal transmission rate of 3.54% which are in line with our study (18). Retrospective follow up studies done in Diredawa (2005–2013) and Jimma (2010–2012), Ethiopia reported a rate of MTCT to be 15.7% and 17%, respectively (19,20) and the major contributors were home delivery, absence of maternal ART and absence of infant ARV prophylaxis. The rate of transmissions in both studies were higher than our report which is due the fact that those studies were done before the implementation of Option B+ which showed significant reduction of HIV infection among HIV exposed infants from different reports. A reduction in the LTFU rate was reported from Kenyan governmental Hospitals from 36% to 22% which is explained by the introduction of Option B+ and strengthening of PMCT uptakes (21,22). This rate is significantly higher than ours (9.4%) which might be due to the strong tracing system in our study area where active tracing has been practiced in the community.

Worldwide significant reductions in rate of HIV infection and LTFU are being observed due to the implementation of strong PMTCT programs (23–26). This study showed us a higher rate of HIV-infection among HIV-exposed infants implying that further studies at different sites with wider range should be done and the overall care of HIV-exposed infants in the hospital should be reevaluated and implementing strong family-centered approach should be done to increase paternal involvement in the care.

Our study was done in a single center with small sample size; findings could have been stronger if it was done at multiple sites with higher sample size.

Strengthening the PMCT activity by advocating antenatal care and institutional deliveries especially in rural areas, defaulter tracing and male involvement can decrease the rates of transmission and LTFU. Our study concluded that the rate of MTCT (3.6%) is still higher than the WHO standard of 2% by 2020 and living in rural areas, developmental failure in infants and unknown fathers' HIV status were significantly associated with the incidence of HIV infection among HIV exposed infants.
